# Impact of Host Cell DNA and Chromatin on Virus‐like Particle Analysis by Light Scattering in Asymmetrical Flow Field‐flow Fractionation

**DOI:** 10.1002/jssc.70313

**Published:** 2025-10-31

**Authors:** Johanna Bacher, Leo A. Jakob, Tomas Mesurado, Narges Lali, Alexander Zollner, Alois Jungbauer, Patricia Pereira Aguilar

**Affiliations:** ^1^ acib ‐ Austrian Centre of Industrial Biotechnology Vienna Austria; ^2^ Department of Biotechnology and Food Science BOKU University, Institute of Bioprocess Science and Engineering Vienna Austria

## Abstract

The development of virus‐like particle (VLP) production processes is often constrained by the extensive number of analytical methods required for their quantification and characterization, as well as the significant labor demands associated with these techniques. Asymmetrical flow field‐flow fractionation (AF4) coupled with in‐line detectors, such as ultraviolet (UV) and multi‐angle light scattering (MALS), presents a promising label‐free and rapid approach to simultaneously assess the quantity and quality of VLP samples. While AF4‐MALS has been widely applied for bionanoparticle characterization and quantification in final products and process development, the influence of host cell‐derived impurities on the outcome of the analysis remains underexplored.

This study investigates the impact of host cell‐derived impurities, particularly host cell DNA and chromatin, on AF4‐MALS‐DLS analysis of both unpurified and purified VLP samples, using HIV‐1 gag VLPs produced in CHO cells as a model system. Our results demonstrate that DNA, chromatin, and VLPs can co‐elute due to their overlapping size distribution, which, if overlooked, may lead to imprecise determination of VLP concentrations in early process samples and inaccurate yield calculations at later stages. Nevertheless, for total particle quantification, AF4‐MALS was shown to be a suitable surrogate for nanoparticle tracking analysis, as the 90° light scattering peak area exhibited a strong linear correlation with total particle concentration. This substitution enables faster sample processing and reduces sample volume requirements.

Additionally, our findings highlight the importance of particle concentration and method parameter selection, particularly the detector flow rate, when characterizing samples based on hydrodynamic radius (R_hyd_). Underestimation of R_hyd_ due to high detector flow rates was proposed as the possible explanation for the higher‐than‐expected shape factors obtained for VLPs. These results emphasize the need for further optimization of AF4 methods to improve the separation of VLPs from host cell impurities and to ensure reliable characterization of bionanoparticles in complex mixtures.

## 1 Introduction

Enveloped virus‐like particles (eVLPs) are an increasingly valuable class of biological therapeutics, with application in therapeutic vaccines and drug delivery systems [1, 2, 3]. During eVLP production, apart from the recombinantly produced eVLPs, cells also release other extracellular vesicles, such as exosomes, microvesicles, and apoptotic bodies. Like eVLPs, extracellular vesicles are covered by a lipid bilayer derived from the host cell membrane and are therefore difficult to separate with high resolution [4, 5, 6]. Besides that, cell lysis during production results in the release of host cell DNA and chromatin into the culture supernatant (SN). These different particle populations (including chromatin fragments) can be similar in size and shape, making the accurate quantification of eVLP particles in the cell culture SN very challenging [5]. In contrast to live viruses, VLPs are non‐infectious, and therefore infectivity‐based assays are unsuitable [7]. Instead, process samples are characterized using methods that assess the total particle concentration and size distribution. Those are often combined with protein characterization assays. Particle quantification based on light scattering spares the need to establish an antigen‐based calibration curve and prior in‐depth particle characterization. Total particle quantification is often performed using nanoparticle tracking analysis (NTA) [8, 9].

NTA is an established process analytical method for measuring particle size distribution and concentration of process samples containing biological nanoparticles in solution. The particles in the sample scatter light when they are illuminated by a laser, and a camera records the trajectory of the individual particles in solution. Nanoparticles, such as eVLPs, are subject to random motion in suspension, known as Brownian motion. The mean square displacement of a particle's motion, based on Brownian motion, is related to the diffusion coefficient (*D_t_*) of the particle. Using the Stokes‐Einstein equation, it is possible to calculate the size of the particle, expressed as hydrodynamic diameter (*d*), from the diffusion coefficient with solvent viscosity (η), absolute temperature (*T)* in *K*elvin, and the Boltzmann constant (*K_B_*). In NTA, particle concentration is measured directly by counting the individual particles observed during the recorded video and relating them to the known analyzed volume. Since all particles are counted as equals, at early process stages, the presence of host cell chromatin and extracellular vesicles might lead to a systematic over‐quantification of eVLPs [10, 11, 12].

To improve particle quantification and characterization in complex mixtures, separation techniques such as size exclusion chromatography (SEC) and asymmetrical flow field‐flow fractionation (AF4) are employed prior to analysis with light‐scattering‐based methods, such as multi‐angle light scattering (MALS) and dynamic light scattering (DLS) [13, 14, 15, 16]. For example, Steppert et al. showed that SEC‐MALS can be used to quantify HIV‐1 gag VLPs and compared the results directly to those obtained using NTA [14]. While a linear correlation was observed between the two methods, particle concentrations determined by SEC‐MALS were approximately 1‐log lower than those measured by NTA. The authors suggest that this discrepancy may arise from the assumptions required by the mathematical models used for particle size and concentration calculations using MALS data, such as the particles being monodisperse, homogeneous, spherical, and having a known refractive index. Additionally, the authors propose that particle aggregation or loss due to nonspecific binding to the stationary phase could contribute to the lower concentration observed. Furthermore, it is important to note that column frits may filter out larger particles, preventing them from reaching the stationary phase or the detectors. Finally, it must be considered that VLPs elute from the SEC column in the void volume, along with other particles of similar or larger size, since all are excluded from the resin pores. Alternatively, AF4 can be used as a separation technique prior to analysis by light scattering [13, 17, 18, 19, 20, 21, 22, 23, 24]. AF4 separates particles based on their hydrodynamic size and diffusion properties, as they flow through a thin channel with a semi‐permeable membrane. As liquid flows through the channel, particles are pushed towards the membrane by a perpendicular cross‐flow field. Smaller particles diffuse farther away from the membrane, reaching regions of faster laminar flow (due to the parabolic velocity profile), while larger particles remain closer to the membrane and travel more slowly. Therefore, contrary to the SEC, in AF4, smaller particles elute first and larger particles elute later. This size‐dependent interplay between cross‐flow and diffusion enables AF4 to separate a wide range of nanoparticles without the need for a stationary phase. VLPs derived from non‐enveloped viruses, such as murine polyomavirus and adeno‐associated virus, have been separated by AF4 and characterized by MALS [20, 25, 26]. Chuan et al. demonstrated that the use of AF4‐MALS allowed for a more detailed characterization of murine polyomavirus‐like particles produced in insect cells compared to traditional techniques such as transmission electron microscopy (TEM) or DLS [20]. However, some particle populations and larger aggregates remained unidentified, highlighting the need for further analytical characterization.

Also enveloped viruses, such as bacteriophage Φ6 and influenza A and B viruses, have been analyzed with AF4‐MALS [16, 21]. Lampi et al. used AF4 as a method for Φ6 purification, obtaining higher recoveries of infectious virus and comparable purities to those obtained by PEG‐precipitation and ultracentrifugation [27]. In addition, a combination of AF4 and MALS/DLS allowed simultaneous Φ6 quantification and particle size determination. The use of light scattering detectors also revealed larger complexes eluting after the virus peak, which were not previously identified when using ultraviolet (UV) [27]. However, those larger particles were not further characterized or identified. Wei et al. demonstrated a good correlation between total influenza virus counts measured using AF4‐MALS, PCR, and TEM, highlighting the utility of AF4 for virus quantification and characterization [16]. However, these studies do not clearly address the impact of host cell‐derived impurities on AF4‐MALS results or how this affects the applicability of the technique for process development.

VLP analysis by AF4‐MALS is performed to determine both particle concentration and size distribution. MALS determines particle size (radius of gyration, r_g_) and count by measuring the angular dependence of the intensity of scattered light and fitting it to light scattering formalisms or shape‐specific models. For particles up to approximately 100 nm, the Rayleigh‐Gans‐Debye approximation is typically applied, while the Mie theory is used for larger particles [28, 29]. However, as mentioned earlier, certain assumptions are required when applying these models to determine particle counts, including the assumption of a monodisperse sample, a known refractive index, and a known particle shape. Moreover, Plavchak et al. recently highlighted the importance of selecting the appropriate models and refractive index values for bioparticles to ensure accurate quantification [29].

Given the presence of diverse particle populations in early process samples during eVLP production, such as cell culture supernatants, it is essential to evaluate the impact of host cell‐derived impurities on the results obtained from AF4‐MALS analysis for eVLP characterization and quantification. To the best of our knowledge, this topic has been insufficiently addressed in the literature. Therefore, this study aims to investigate the influence of critical host cell‐derived impurities, specifically host cell DNA and chromatin, on the AF4‐MALS‐DLS analysis of eVLPs. This method enables insights into the particle's shape besides size distribution due to the simultaneous measurement of hydrodynamic radius by DLS in the MALS detector [30]. For that, HIV‐1 gag VLPs produced in CHO cells were used as a model eVLP [31, 32]. Unpurified and purified samples were analyzed. HIV‐1 gag VLPs were purified by previously developed methods using restricted access and heparin affinity chromatography [5, 6, 33]. Particle count obtained by AF4‐MALS was directly compared to NTA measurements. Additionally, double‐stranded DNA (dsDNA) was quantified by PicoGreen assay, and chromatin presence was assessed by Western blot.

## 2 Material and Methods

### 2.1 Chemicals and Materials

Filters were acquired from Thermo Fisher Scientific (Waltham, MA, USA). Chromatographic resins were acquired from Cytiva (Uppsala, Sweden). The AF4 membranes were acquired at Wyatt Technology (Santa Barbara, CA, USA). SDS PAGE gels and LDS sample buffer were acquired from Invitrogen (Carlsbad, CA, USA). Protein markers, reagents, and gel stains were acquired from Bio‐Rad (Hercules, CA, USA) or Thermo Fisher Scientific (Waltham, MA, USA). All chemicals were of analytical grade and acquired from Sigma Aldrich (St. Louis, MO, USA) or Merck (Darmstadt, Germany). Antibodies were acquired at Abcam (Cambridge, United Kingdom) or Sigma Aldrich (St. Louis, MO, USA). The dsDNA assay was acquired from Invitrogen (Carlsbad, CA, USA).

### 2.2 HIV‐1 Gag VLP Production and Purification

CHO cell culture SN was provided by Icosagen (Tartumaa, Estonia). The expression of VLPs based on HIV‐1 gag protein was done in a pQMCF expression vector expressing HIV‐1 gag protein under the control of the CMV promoter as previously described by Steppert et al. [32]. Production of HIV‐1 gag VLPs was confirmed by Western blot analysis detecting HIV‐1 p24. After production of the HIV‐1 gag VLPs, cells were removed by centrifugation (1000 g, 30 min), and 0.01% NaN3 was added.

The CHO cell culture SN was filtered with a 0.8 µm Cellulose Nitrate Nalgene Rapid‐Flow Sterile Disposable Filter Unit (Thermo Fisher Scientific, Waltham, MA, USA) in order to remove cell debris and large impurities.

The purification of the HIV‐1 gag VLP material consisted of a pre‐purification using the restrictive access media, Capto Core 700, followed by Heparin affinity chromatography using Capto Heparin (both, Cytiva, Uppsala, Sweden), previously described in more detail by Zollner et al. [33]. All columns were packed in HiScale 16/20 column housings (Cytiva, Uppsala, Sweden), and the experiments were performed with an ÄKTA Pure 25 instrument equipped with a sample pump S9 and a fraction collector F9‐C (Cytiva, Uppsala, Sweden). The Unicorn software 6.4.1 was used for data collection. UV signals at 280 and 260 nm, as well as conductivity, were monitored online. Particle elution was monitored by MALS with a DAWN 8 detector by Wyatt Technology (Santa Barbara, California) coupled to the chromatographic system. The detector's mV signal at 10% laser power, detector 5 (90° angle), and an output slope of 0.2 were directly displayed and recorded in the Unicorn software.

### 2.3 Asymmetrical Flow Field‐Flow Fractionation

The AF4 Eclipse was connected to an Agilent 1200 High‐Performance Liquid Chromatography system equipped with a multichannel gradient quaternary pump (Agilent, Santa Clara, USA). The Eclipse AF4 aqueous phase long channel was lined with a 350 µm spacer and a polyethersulfone (PES) membrane with a 10 kDa cut‐off (Wyatt Technology, Santa Barbara, CA, USA) was used. The mobile phase was 1x phosphate‐buffered saline (PBS), pH 7.2 (0.1 µm filtered), and VLP samples were injected undiluted or diluted in the same buffer as the mobile phase before injection into the AF4 system. The detector flow was kept constant at 0.5 mL/min. First, the VLP samples were injected by hydrodynamic relaxation at a flow rate of 0.1 mL/min and a crossflow of 1.2 mL/min. After 5 min at a constant flow rate, a linear crossflow gradient was held for 15 min until a crossflow of 0.2 mL/min was reached. In the next 20 min, the crossflow was set to zero to elute highly retained analytes from the channel. Samples were collected during the whole run in 1 mL fractions. System control and data acquisition were performed in OpenLab CDS ChemStation (Agilent, Santa Clara, USA) and ASTRA 7.3.3 software (Wyatt, Santa Barbara, CA, USA). The light scattering peak areas as well as the particle characteristics were measured in a DAWN 8 system equipped with a Wyatt QELS module for dynamic light scattering.

### 2.4 Particle Concentration and Size Distribution

NTA was used to analyze particle concentrations and particle size distribution using a NanoSight NS300 (Malvern Instruments, Worcestershire, UK) with a blue laser module working at a wavelength of 488 nm. Samples were serially diluted using particle‐free water and measured at a concentration of 20–80 particles per video frame. The samples were measured in light scattering mode, and three videos were recorded per sample, each measured in three different dilutions, resulting in nine recorded videos of 60 s per sample. The camera level as well as the focus were manually adjusted prior to each measurement. The recorded videos were analyzed in the NanoSight NTA software version 3.2 (Malvern Instruments, Worcestershire, UK).

### 2.5 Sodium Dodecyl Sulphate‐Polyacrylamide Gel Electrophoresis and Western Blotting

Sodium dodecyl sulphate‐polyacrylamide gel electrophoresis (SDS‐PAGE) was performed in an X‐cell SureLock Mini‐Cell electrophoresis chamber (Invitrogen, Carlsbad, CA, USA), using NuPAGE Bis/Tris 4%–12% gels (Invitrogen, Carlsbad, CA, USA) and reduced MES‐SDS running conditions at 200 V and 400 mA for 45 min. The analyzed samples were prepared with NuPAGE LDS sample buffer (Invitrogen, Carlsbad, CA, USA) and reduced at 99°C for 15 min in the presence of 182 mM dithiothreitol (DTT). The Precision Plus Protein Unstained marker (Bio‐Rad, Hercules, CA, USA) was used as a molecular weight marker for SDS‐PAGEs. For the Western blot analysis, SeeBlue Plus2 Pre‐stained Protein Standard (Thermo Fisher Scientific, Waltham, MA, USA) was used. Protein bands in the gels for the SDS‐PAGE were stained with Flamingo Fluorescent Protein Gel Stain (Bio‐Rad, Hercules, CA, USA).

For Western blot analysis, proteins were transferred from the gel to a 0.2 mm nitrocellulose membrane using the Trans‐Blot Turbo Transfer System (Bio‐Rad, Hercules, CA, USA) according to the manufacturer's instructions. Membranes were blocked overnight with 3% w/v bovine serum albumin (BSA) in PBS‐T (0.1% w/v Tween‐20 in PBS). The membranes were incubated with the following primary antibodies diluted 1:1000 in PBS‐T containing 1% w/v BSA for 2 h: mouse monoclonal antibody against HIV‐1 p24 (Abcam, Cambridge, United Kingdom) and rabbit monoclonal antibody against Histone H3 (Abcam, Cambridge, United Kingdom). Then, the membranes were incubated with the respective anti‐mouse or anti‐rabbit IgG conjugated with alkaline phosphatase (Sigma Aldrich, St. Louis, MO, USA), diluted 1:1000 in PBS‐T with 1% w/v BSA for 1 h. Premixed BCIP/NBT solution (Sigma Aldrich, St. Louis, MO, USA) was used as a substrate for visualizing the respective bands on the blots.

### 2.6 Total Protein and dsDNA Content

The total protein content was determined by the Bradford assay in a 96‐well plate format using a Coomassie blue G‐250–based protein dye reagent (Bio‐Rad Laboratories, Hercules, CA, USA) in accordance with the manufacturer's instructions. Calibration curves were obtained using BSA standards diluted in TE buffer.

The Quant‐iT PicoGreen dsDNA Assay Kit (Invitrogen, Carlsbad, CA, USA) was used to determine the content of dsDNA in a 96‐well format according to the manufacturer's instructions.

The measurement of both assays was carried out using a Spark microplate reader (Tecan, Männedorf, Switzerland).

## 3 Results and Discussion

### 3.1 Characterization of HIV‐1 Gag VLPs in Unpurified Samples by AF4‐MALS

**FIGURE 1:**
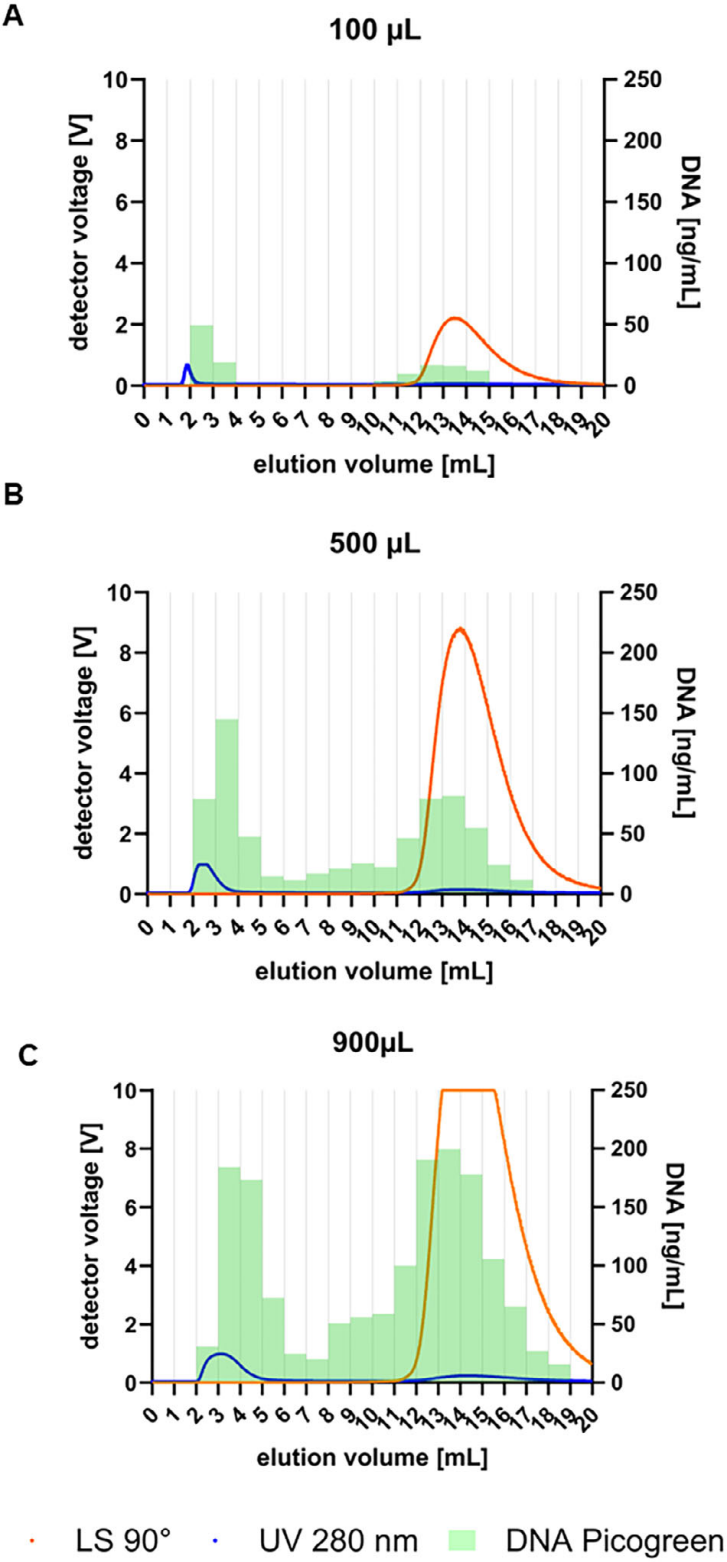
Fractograms of clarified cell culture supernatant (SN) containing HIV‐1 gag virus‐like particles (VLPs) injected into the asymmetrical flow field‐flow fractionation (AF4) system at injection volumes of 100 µL (A), 500 µL (B), and 900 µL (C). Lines show the in‐line measured LS90° (orange) and UV280nm (blue), and bars represent the off‐line measured double‐stranded DNA (dsDNA) concentrations in ng/mL (determined by PicoGreen assay).

**FIGURE 2:**
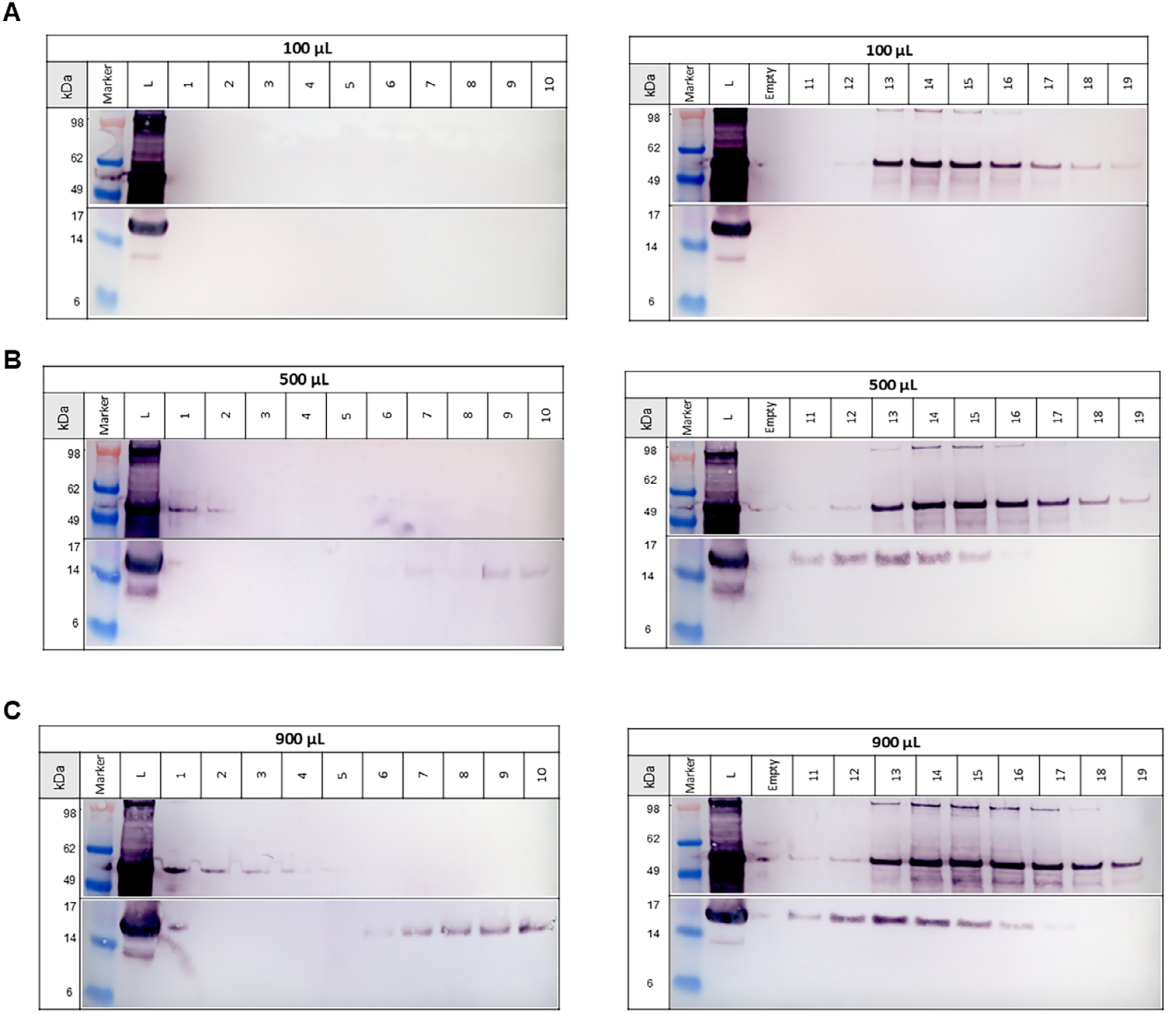
Western blot analysis of collected fractions from the asymmetrical flow field‐flow fractionation (AF4) runs with injection volumes of 100 µL (A), 500 µL (B), and 900 µL (C). Western blot against p24 protein for detection of HIV‐1 gag virus‐like particles (VLPs) (∼55 kDa, top) and against H3 histone protein for detection of chromatin (∼15 kDa, bottom). The sodium dodecyl sulphate‐polyacrylamide gel electrophoresis (SDS‐PAGE) gels were loaded with a protein ladder (Marker), the clarified cell culture supernatant containing HIV‐1 gag VLPs (L), as well as the AF4 collected elution fractions [1, 2, 3, 4, 5, 6, 7, 8, 9, 10, 11, 12, 13, 14, 15, 16, 17, 18, 19].

CHO cell culture harvest containing HIV‐1 gag VLPs was clarified by 0.8 µm filtration to remove cell debris. The clarified cell culture SN containing 2.55 × 10^11^ (± 0.31 × 10^11^) total particles/mL, 686 (± 138) µg/mL total protein, and 2678 (± 168) ng/mL dsDNA, was used to investigate the application of the AF4 system for separation of different bionanoparticle populations and characterization of eVLPs in complex mixtures by MALS and DLS. Specifically, the impact of the presence of host cell dsDNA and chromatin on the eVLP quantification and characterization was investigated. Three different injection volumes (100, 500, and 900 µL) were used to investigate the elution profiles of protein, DNA, and particles after injection in the AF4 system. Figure 1 shows the fractograms of the different injections of SN containing HIV‐1 gag VLPs. In addition to the online UV280 and LS signals, collected fractions were analyzed by PicoGreen assay, and the off‐line measured dsDNA concentration of each fraction is depicted in the fractogram (Figure 1, green bars). For all three tested injection volumes, the first elution peak (starting at approximately 2 mL) shows a UV280 signal but no LS signal, indicating the elution of small species but no particles. PicoGreen assay results of the collected fractions confirmed the elution of dsDNA in this phase of the AF4 method (Figure 1, green bars), in which a crossflow of 1.2 mL/min was used. For all collected fractions, total protein content was below the limit of quantification of the Bradford assay (12.5 µg/mL), and protein visualization using SDS‐PAGE was only possible for the higher injection volumes (500 and 900 µL) due to sample dilution in the AF4 channel (Figure S1 in Supplementary Material). Particle elution was only observed in the second peak, starting from approximately 11–12 mL (Figure 1, LS signal), when the crossflow was set to zero, revealing high particle retention for the selected AF4 method. For the higher injection volumes (500 and 900 µL), Western blot analysis of the fractions collected in the second peak revealed not only the presence of p24, indicating the presence of HIV‐1 gag VLPs, but also the presence of histone H3 proteins, indicating the co‐elution of HIV‐1 gag VLPs and chromatin (Figure 2). The fact that this was not observed at the 100 µL injection is due to the sample dilution that occurs on the AF4 channel (injection of 100 µL and peak distributed over 10 mL).

In addition to the Western blot results, the PicoGreen assay revealed dsDNA elution in the second peak, supporting the hypothesis of chromatin elution. Although both species partially co‐eluted in the second peak, Western blot results revealed that the selected AF4 method allowed partial separation of eVLPs and chromatin, as the H3 histone protein was detected in earlier fractions while the p24 protein was detected in later fractions (Figure 2B,C). However, it is important to note that chromatin fragments exhibit a wide size distribution, ranging from the “10‐nm chromatin fiber”, small nucleosomes wrapped around spaced core histones, approximately 10 nm in size, to larger chromatin fragments up to 1000 nm [34]. This broad size range may lead to the inevitable co‐purification of certain chromatin fragments with eVLPs when size‐based separation methods are employed. These results suggest that further method optimization, such as cross‐flow gradient optimization, could improve the separation of eVLPs and chromatin fragments and potentially enable the use of AF4 at a semi‐preparative scale for the preparation of highly pure bionanoparticles. Alternatively, to enhance bionanoparticle purity in AF4 measurements and improve the accuracy of MALS for quantification, sample pre‐treatment with a salt‐active endonuclease, combined with the selection of a membrane with a higher molecular weight cut‐off, could facilitate more effective chromatin removal [4, 35].

### 3.2 Correlation Between LS90° and Particle Concentration in Clarified Cell Culture S

Although complete separation of different bionanoparticles, such as VLPs and chromatin, could not be achieved with the selected AF4 method, the correlation between the light scattering measured by the 90° angle detector (LS90°) and the total particle concentration determined by NTA was investigated for clarified cell culture SN containing HIV‐1 gag VLPs.

**FIGURE 3:**
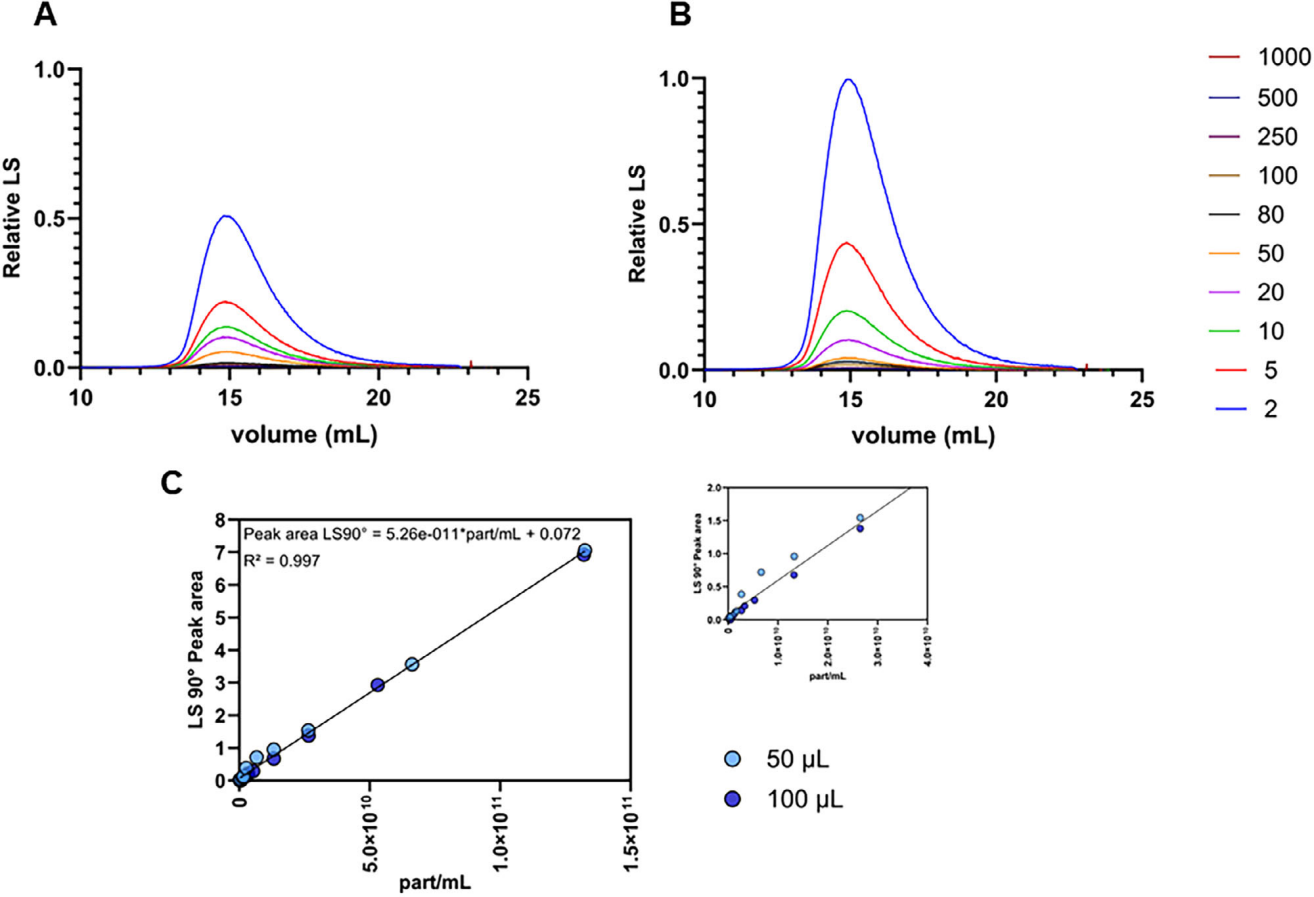
Asymmetrical flow field‐flow fractionation (AF4) fractograms of supernatant (SN) containing HIV‐1 gag virus‐like particles (VLPs) at different particle concentrations (dilution factors: 2, 5, 10, 20, 50, 80, 100, 250, 500, and 1000) injected at a volume of 50 µL (A) and 100 µL (B). Correlation of the 90° light scattering peak area (LS90°) with the total particle concentration measured by nanoparticle tracking analysis (NTA) (C).

Different dilutions of SN were injected into the AF4 system using two injection volumes: 50 µL (Figure 3A) and 100 µL (Figure 3B). The obtained fractograms demonstrated that, for the same injection volume, the LS90° signal decreased with increasing dilution, as expected, due to the reduction in particle concentration and the corresponding decrease in scattered light intensity. No changes in the peak profile were observed. Furthermore, when the same total particle amount was injected at different volumes (and dilutions), the LS90° peak area remained consistent, indicating that the injection volume does not influence the results (within the tested range). Since particle separation was not achieved with the selected AF4 method and NTA measures all particles present in the sample, the obtained LS90° peak area was compared with the total particle concentration determined by NTA (Figure 3C). A linear correlation (with R^2^ of 0.997) between LS90° and NTA particle concentration could be observed within the tested range (2.6 × 10^8^ and 1.3 × 10^11^ particles/mL, determined by NTA). Considering the linear correlation between the two methods, AF4‐MALS, using only the LS90° peak area and a correlation curve, can be used to replace NTA measurements and accelerate process development, as it reduces the required operator efforts.

### 3.3 Particle Size Determination by AF4‐MALS‐DLS in Clarified Cell Culture SN

**FIGURE 4:**
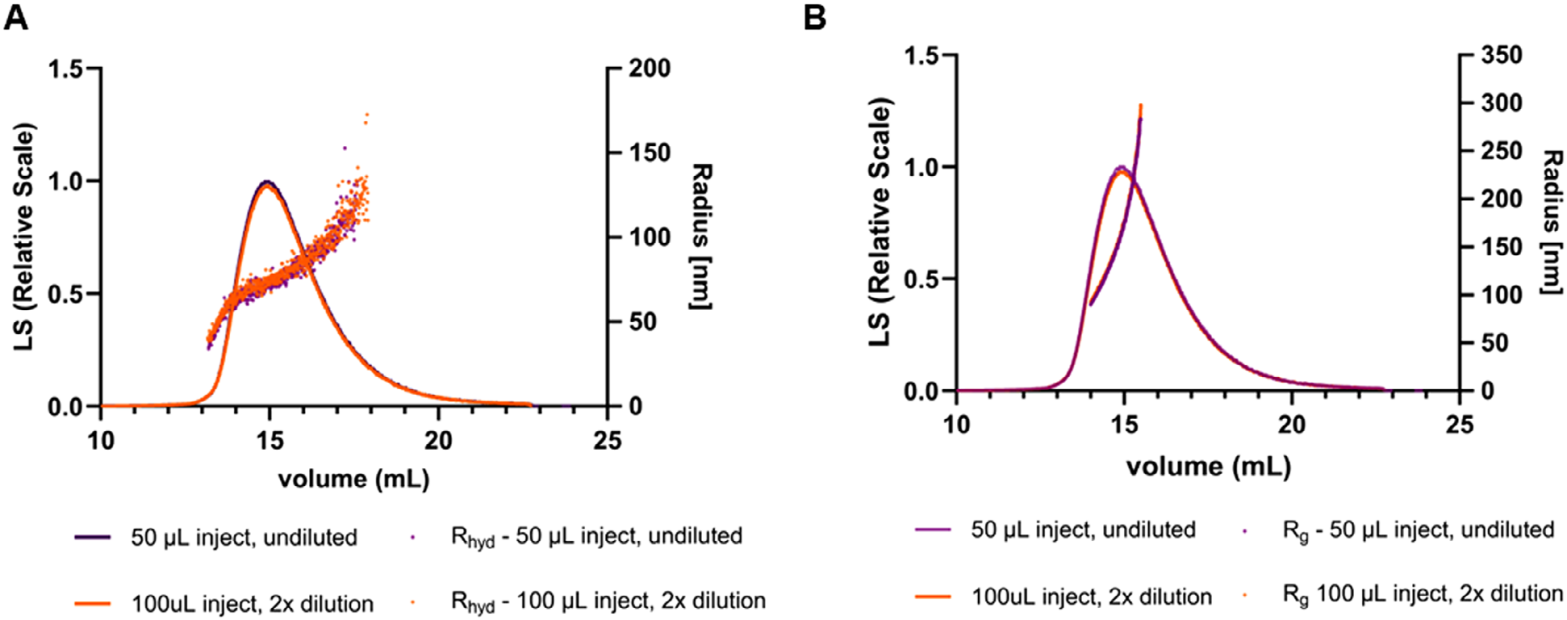
Fractograms of 50 and 100 µL injections of supernatant (SN) containing HIV‐1 gag virus‐like particles (VLPs). The LS90° signal is shown in both fractograms together with the calculated hydrodynamic radius, R_hyd_, from the dynamic light scattering (DLS) data (A) and the radius of gyration, R_g_, from the multi‐angle light scattering (MALS) data (B).

In addition to particle concentration, MALS and DLS data collected during the AF4 run were used to determine both the radius of gyration (R_g_​) and the hydrodynamic radius (R_hyd_​) of the particles. This was done for both injection volumes (50 and 100 µL injection). In order to have the same number of injected particles, a two‐fold dilution was performed for the 100 µL injection. Figure 4 shows the LS90° signal of the second peak in the fractogram together with the calculated radii (Figure 4 A, R_hyd_ and Figure 4B, R_g_). Both R_g_ and R_hyd_ increased with the elution volume, showing the sample polydispersity. As expected, smaller particles with higher diffusivity eluted earlier than larger particles with lower diffusivity. Interestingly, a steeper increase in radius was observed for R_g_ than for R_hyd_. While R_g_ increased from approximately 100 to 300 nm (Figure 4B), R_hyd_ varied from approximately 50 to 125 nm (Figure 4A).

**FIGURE 5:**
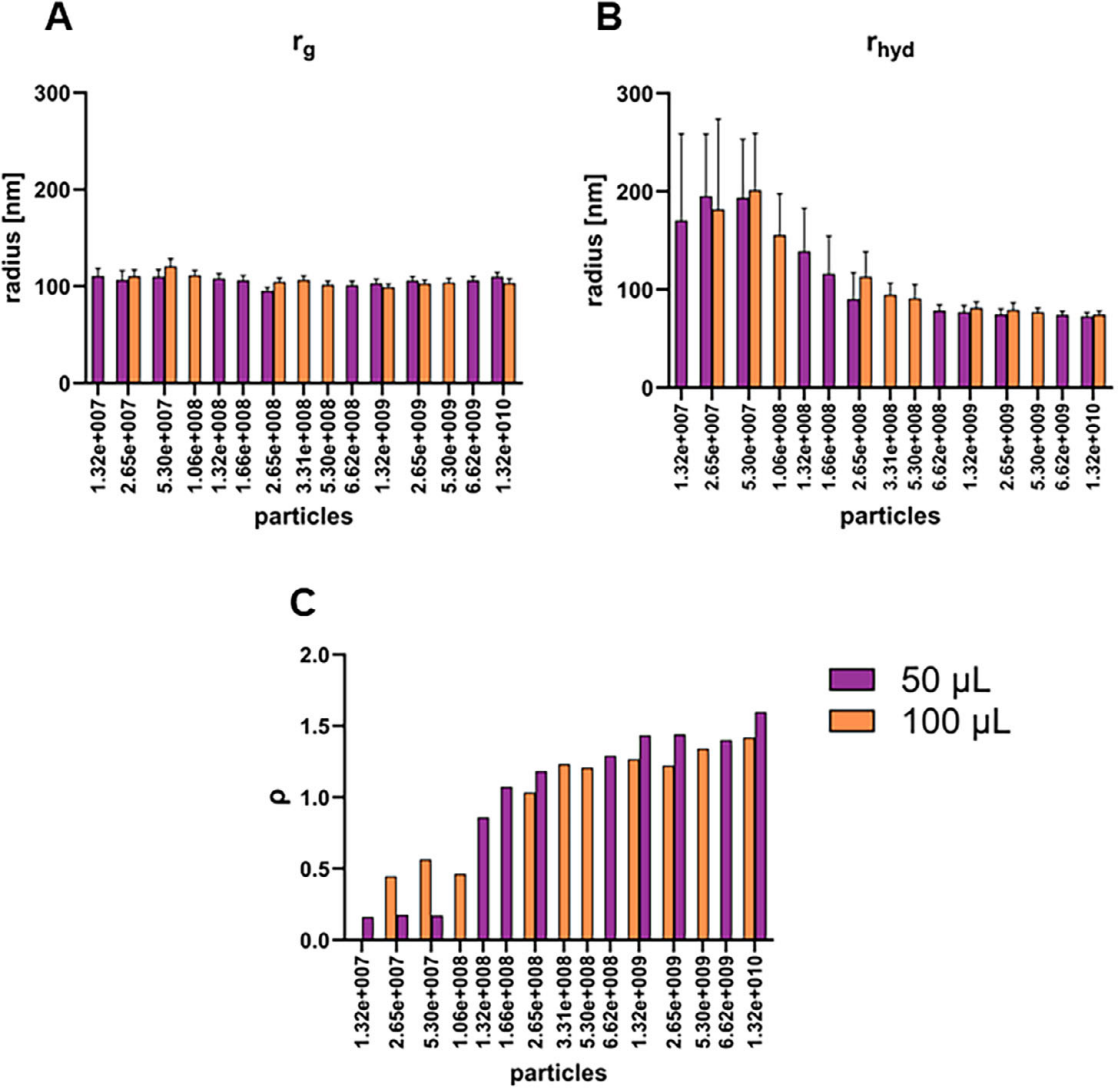
Radii analysis showing R_g_ (A) and R_hyd_ (B) from injections of different volumes and dilutions of supernatant (SN) containing HIV‐1 gag virus‐like particles (VLPs). The shape factor ρ (C) shows the ratio: $\frac{R_{g}}{R_{hyd}}$.

The ratio between R_g_ and R_hyd_ can be used to estimate the molecular shape, also known as the shape factor or ρ [17, 30]. The shape factor reflects the mass distribution of a particle in relation to its hydrodynamic footprint in solution. Typical values range from 0.775 for a solid sphere, 1.5‐1.8 for a random coil polymer, ∼1 for a hollow sphere (depending on the shell thickness), and ≥2.0 for rods [36]. Considering the R_g_ and R_hyd_ values obtained for the LS90° peak maximum of each injected particle amount (Figures 5A and 5B, respectively), the shape factor was calculated (Figure 5C). The results indicate that the R_g_ value at peak maximum remained constant at approximately 100 nm, regardless of the injected particle amount. In contrast, the R_hyd_ values varied with different injected amounts of the same sample. These findings highlight the importance of injecting a sufficient amount of particles to ensure accurate determination of the hydrodynamic radius and, consequently, an accurate calculation of the shape factor. For the higher particle amounts (> 6.6 × 10^8^), an R_hyd_ of approximately 70 nm was obtained, resulting in shape factors ranging from 1.2‐1.4. Considering the structure of the HIV‐1 gag VLP as a hollow sphere, a shape factor of 0.9‐1.0 would be expected. Shape factors in the range of 1.2‐1.4 are typically associated with more elongated, flexible, fragmented, or aggregated forms. Since the co‐elution of DNA and chromatin was confirmed by off‐line analytical methods, this could explain the higher‐than‐expected shape factor values.

### 3.4 Correlation Between LS90° and Particle Concentration in Purified HIV‐1 Gag VLP and Process Samples

**FIGURE 6:**
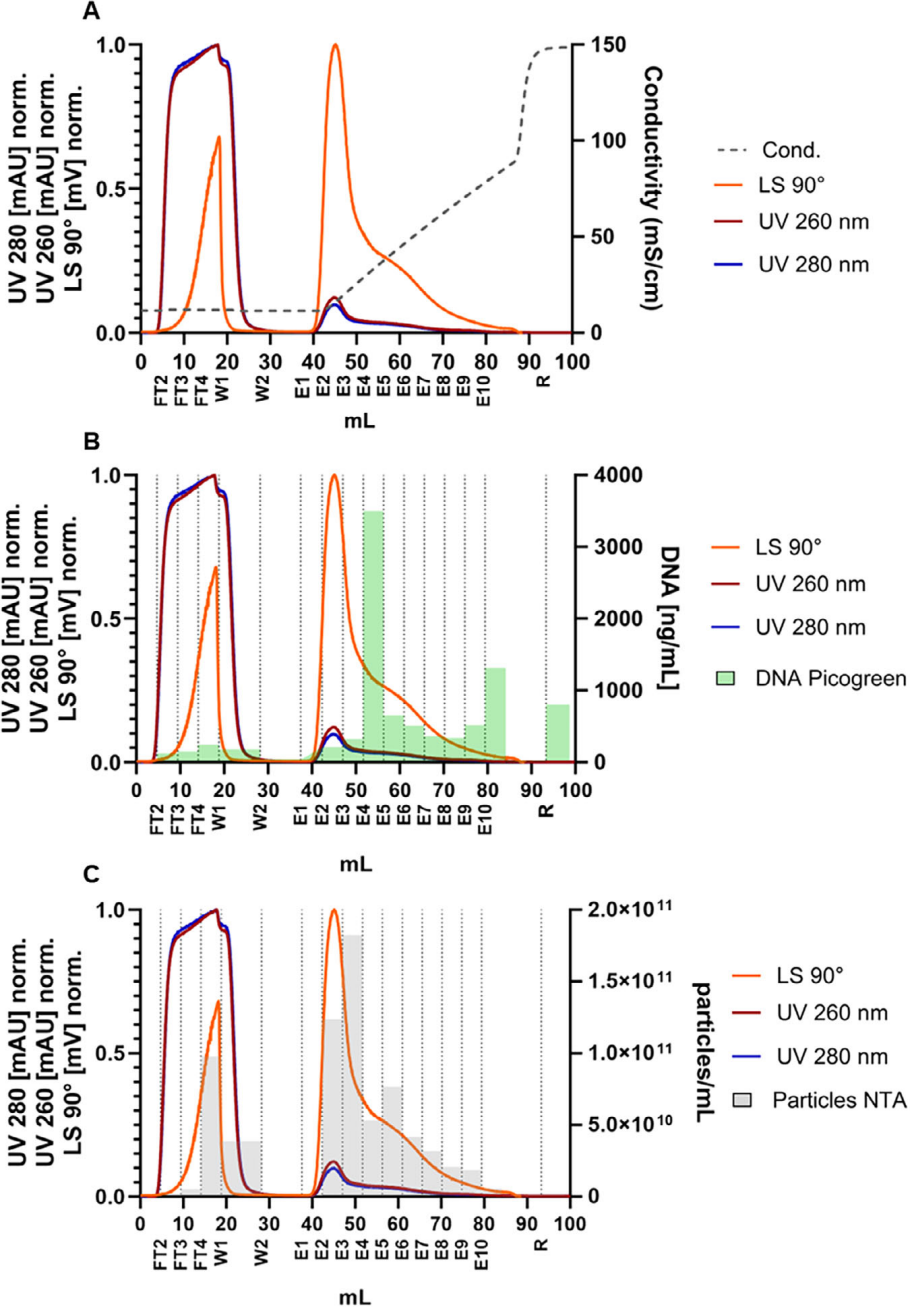
Chromatogram of Capto Heparin purification step. The chromatograms show the in‐line measured UV280, UV260, and 90° light scattering signals with conductivity signal (A) and corresponding off‐line measured double‐stranded DNA (dsDNA) concentrations in ng/mL measured by PicoGreen assay (B), as well as particle concentrations measured by nanoparticle tracking analysis (NTA) (C). The fractions were collected as flow‐through (FT2–FT4), Wash (W1, W2), Elution (E2–E10), and Regeneration (R).

HIV‐1 gag VLPs were purified from clarified cell culture SN using a combination of flow‐through and heparin affinity chromatography with Capto Core 700 and Capto Heparin resins, respectively [5, 6, 33]. The first chromatographic step, flow‐through chromatography, is used to remove small impurities, smaller than 50 nm, such as host cell protein and dsDNA [37]. In this step, particles larger than 50 nm are collected in the flow‐through during the loading phase. Chromatin fragments released upon cell lysis can span an exceptionally broad size range from small nucleosomes of approximately 10 nm up to highly extended chromatin aggregates reaching 1000 nm [38]. Consequently, initial purification steps relying on size‐based exclusion, such as the use of Capto Core 700, are unable to efficiently remove all chromatin contaminants. Capto Core 700 excludes molecules above ∼700 kDa (roughly corresponding to particles >50 nm) and retains smaller soluble impurities through internal ligands, resulting in the co‐elution of VLPs and large chromatin fragments in the flow‐through [39]. Therefore, the application of a separation method, such as AF4, becomes necessary to further resolve these co‐eluting particles based on their diffusion and hydrodynamic properties before analysis by MALS and DLS [4, 38, 40]. In the following step, the collected flow‐through fraction is used to load the heparin affinity resin, in which different particle populations are separated [5, 6, 33, 35]. Results of the heparin affinity chromatography are shown in Figure 6.

**FIGURE 7:**
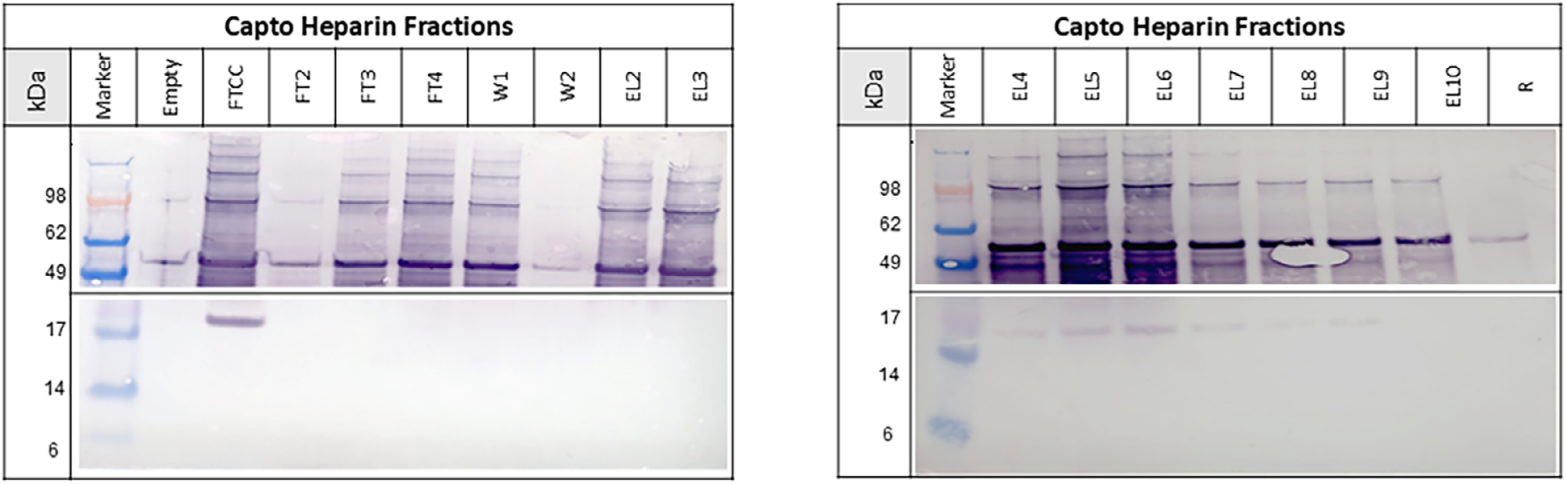
Western Blot analysis showing fractions of the Capto Heparin purification run blotting HIV‐1 gag virus‐like particle (VLP) antigen p24 and histone protein H3.

Although particle breakthrough could be observed during the loading phase (Figure 6, LS90°, FT3–FT4), the majority of the loaded particles bound to the resin, with only 16% of particles found in the flow‐through (Table 1, FT2–FT4). Bound HIV‐1 gag VLPs mainly eluted in the first elution peak, representing 47% of the total loaded particles (Figure 6 and Table 1, E2–E3). In the second elution peak (Figure 6, E4–E9), dsDNA and H3 histone protein were detected (Figure 6 B and Figure 7, respectively), indicating the elution of DNA and chromatin. The absence of H3 histone protein in the first peak (Figure 7, E2–E3) and the lower amount of dsDNA (Figure 6B, E2–E3) demonstrate the separation of DNA and chromatin from the HIV‐1 gag VLPs, even though baseline separation could not be achieved. Similar results have been previously reported [5, 35].

**TABLE 1:** Particle concentrations of collected fractions from the chromatography runs (measured by nanoparticle tracking analysis [NTA]). The fractions are load of flow‐through chromatography (Load CC), and load (Load CH), flow‐through (FT2–FT4), Wash (W1, W2), Elution (E2–E10) and Regeneration (R) of heparin affinity chromatography.

Sample	Volume [mL]	Concentration [part/mL]	SD [part/mL]	RSD [%]	Recovery [%]
Load CC	16.3	2.1E+11	1.8E+10	8	−
Load CH	16.3	1.9E+11	1.3E+10	7	100
FT2	4.7	9.5E+08	1.2E+08	12	0
FT3	4.7	5.3E+09	5.1E+08	10	1
FT4	4.7	9.8E+10	5.7E+09	6	15
EL2	4.7	1.2E+11	1.9E+10	15	19
EL3	4.7	1.8E+11	1.1E+10	6	28
EL4	4.7	5.3E+10	4.5E+09	9	8
EL5	4.7	7.7E+10	1.5E+10	15	12
EL6	4.7	4.2E+10	7.3E+09	18	6
EL7	4.7	3.2E+10	5.3E+09	17	5
EL8	4.7	2.1E+10	3.6E+09	17	3
EL9	4.7	1.8E+10	2.9E+09	16	3
EL10	4.7	5.7E+09	1.2E+09	21	1
R	18.8	4.0E+08	6.1E+07	15	0
W1	9.4	3.9E+10	4.1E+09	11	12
W2	9.4	5.9E+08	1.0E+08	17	0

**FIGURE 8:**
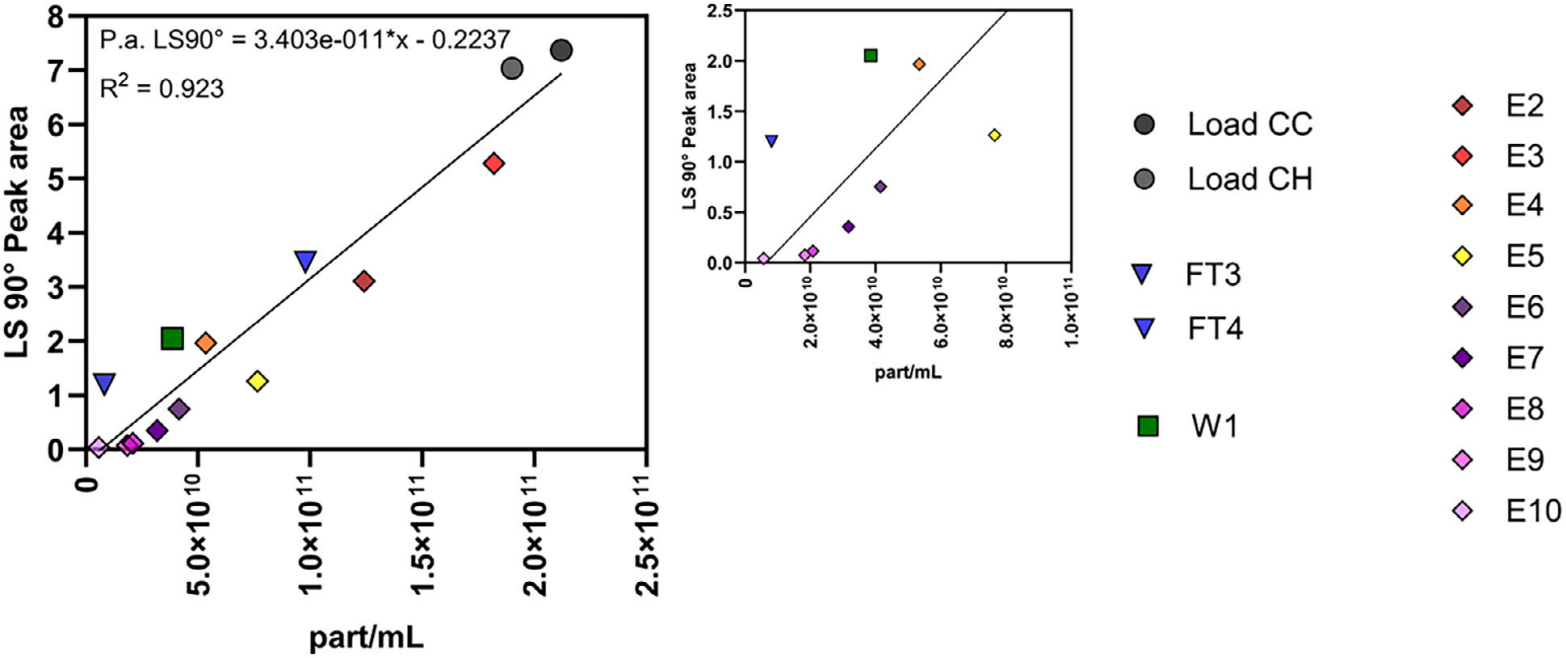
Correlation between the particle concentration measured by nanoparticle tracking analysis (NTA) and the LS90° peak area for selected fractions collected during the chromatography runs. The fractions are load of flow‐through chromatography (Load CC) and load (Load CH), flow‐through (FT3‐FT4), Wash (W1), and Elution (E2–E10) of heparin affinity chromatography.

Fractions collected from the chromatography runs were injected into the AF4‐MALS‐DLS system with an injection volume of 100 µL, and the LS90° peak area was compared to the particle concentration measured by NTA. A linear correlation with an R^2^ of 0.923 was observed when considering all fractions (Figure 8). Interestingly, as discussed in section 3.2, when injecting different dilutions and volumes of the same complex sample (SN containing HIV‐1 gag VLPs, DNA, and chromatin), a stronger linear correlation (R^2^ = 0.997) was obtained (for a particle concentration range of 2.6 × 10^8^ and 1.3 × 10^11^ particles/mL). The light scattering intensity, and consequently the LS90° peak area, depends not only on the particle concentration, but also on the particle size, structure, and the refractive index increment (dn/dc) [28]. As a result, when different particle populations are separated, each population may exhibit a distinct LS90° response, even at the same particle concentration. This variability arises because light scattering is highly sensitive to differences in particle properties, such as their geometry, internal composition, and optical characteristics. Considering this, the presence of impurities in a sample analyzed by AF4‐MALS‐DLS should not be overlooked, particularly if they co‐elute with the target product. This is especially important in bionanoparticle analysis, where co‐produced particles often have overlapping size distributions and are challenging to separate effectively. In order to overcome these issues, efforts should be made to develop novel separation technologies that are not only based on size but also on other molecular features of the bionanoparticles, such as surface charge or affinity to specific ligands.

### 3.5 Particle Size Determination by AF4‐MALS‐DLS in Purified HIV‐1 Gag VLP and Process Samples

**FIGURE 9:**
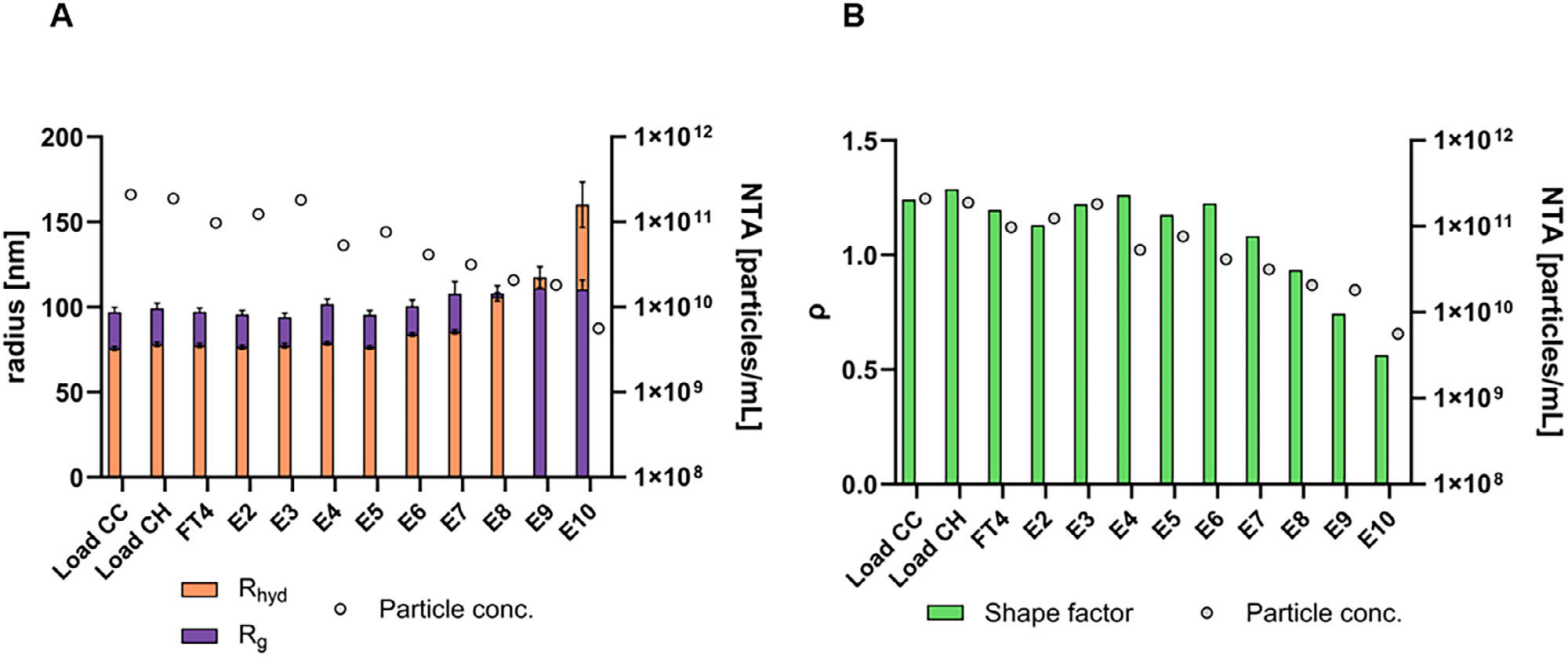
Radii analysis showing R_g_, R_hyd,_ and particle concentration (A), and shape factor ρ (B) for selected fractions collected during the chromatography runs. The fractions are load of flow‐through chromatography (Load CC), and load (Load CH), flow‐through (FT3–FT4), Wash (W1), and Elution (E2–E10) of heparin affinity chromatography. The injection volume was 100 µL.

As for the unpurified samples, MALS and DLS data collected during the AF4 runs of purified samples were also used to determine both R_g_ and R_hyd_ (Figure 9A). HIV‐1 gag VLPs have a particle size of approximately 100–200 nm in diameter [5], which is consistent with the values obtained for both R_g_ and R_hyd_ in fractions E2 and E3, where VLPs are mostly present. Given that VLPs are structurally similar to hollow spheres, shape factors in the range of 0.8‐1.0 would typically be expected. However, the results indicated that for fractions E2 and E3, the shape factor ranged from 1.2 to 1.3. While the obtained results showed a lower R_hyd_ than R_g_, the opposite would have been expected given the structure of the VLPs. One possible explanation is that the selected method conditions may have led to an underestimation of R_hyd_ or an overestimation of R_g_. In fact, Sitar et al. reported that accurate measurements of R_hyd_ by flow DLS are highly dependent on the selected detector flow rate [41]. Their experiments demonstrated that for particles with a radius of 100 nm, accurate R_hyd_ measurements could only be achieved at a detector flow of 0.2 mL/min, while higher flow rates resulted in smaller R_hyd_ values than expected. Considering that our AF4 method used a detector flow rate of 0.5 mL/min, an underestimation of R_hyd_ is hypothesized, which could explain the higher‐than‐expected shape factor. An alternative explanation for the deviation in the shape factor could be the presence of host cell‐derived membrane proteins or glycoproteins on the surface of the VLPs or encapsidated host cell proteins or nucleic acids, which may increase R_g_ relative to R_hyd_, thereby elevating the shape factor. As a result, further considerations regarding the shape factor of other fractions cannot be reliably made, as the observed values may also be influenced by this methodological limitation.

## 4 Conclusion

AF4‐MALS‐DLS has demonstrated significant potential as a versatile tool for the characterization and quantification of VLPs in both unpurified and purified samples. It offers at‐line monitoring capabilities, aligning with PAT's goal of real‐time or near‐real‐time data acquisition, thereby supporting improved process development and control. However, our study revealed that due to overlapping size distributions, host cell‐derived impurities, such as DNA and chromatin, can co‐elute with VLPs during AF4‐MALS‐DLS analysis, potentially leading to overestimation of VLP concentrations and inaccurate yield calculations. Furthermore, the co‐elution of VLPs and chromatin prevents the application of fundamental light‐scattering formalisms for particle concentration determination, as it becomes impossible to precisely define one particle's refractive index and shape.

Nevertheless, the LS90° peak area in AF4‐MALS measurements showed a strong correlation with NTA for total particle quantification, providing a faster and less resource‐intensive alternative for in‐process monitoring. Additionally, AF4‐MALS‐DLS offers valuable insights into critical quality attributes, such as hydrodynamic radius (R_hyd_​), radius of gyration (R_g_), and shape factors, which are essential for ensuring VLP structural integrity. However, method parameter selection, particularly the detector flow rate, was found to be critical, as underestimation of R_hyd_ at higher flow rates likely contributed to higher‐than‐expected shape factors.

These findings underscore the need for further optimization of AF4 methods to improve impurity separation and ensure reliable VLP characterization. Future efforts should focus on refining separation techniques and exploring pre‐treatment strategies to enhance VLP purity and analytical outcomes.

## 5 Use of Artificial Intelligence

During the preparation of this work, the authors used artificial intelligence (ChatGPT OpenAI) in order to enhance readability. Afterwards, the authors reviewed and edited the content as needed and took full responsibility for the content of the publication.

## Author Contributions

**Johanna Bacher**: conceptualization, investigation, writing – original draft, methodology, visualization, data curation, and validation. **Leo A. Jakob**: conceptualization, methodology, and writing – review and editing. **Tomas Mesurado**: investigation and methodology. **Narges Lali**: supervision, conceptualization, methodology, investigation, and validation. **Alexander Zollner**: investigation, methodology. **Alois Jungbauer**: conceptualization, funding acquisition, writing – review and editing, supervision, project administration, and resources. **Patricia Pereira Aguilar**: conceptualization, funding acquisition, writing – review and editing, methodology, project administration, supervision, resources, and validation.

## Conflicts of Interest

The authors declare no conflicts of interest.

## Supporting information




**Supporting File**: jssc70313‐sup‐0001‐SuppMat.docx.

## Data Availability

The data that support the findings of this study are available from the corresponding author upon reasonable request.
