# Supplementary material for: Impact of Host Cell DNA and Chromatin on Virus‐like Particle Analysis by Light Scattering in Asymmetrical Flow Field‐flow Fractionation
Source: J Sep Sci. 2025 Oct 31;48(11):e70313. doi: 10.1002/jssc.70313 (PMC12578362; doi:10.1002/jssc.70313)
Supplement: Supplementary file 1 — Supporting File: jssc70313‐sup‐0001‐SuppMat.docx. [file JSSC-48-e70313-s001.docx]

Impact of host cell DNA and chromatin on virus-like particle analysis by light scattering in asymmetrical flow field-flow fractionation

*Johanna Bacher, Leo A. Jakob, Tomas Mesurado, Narges Lali, Alexander Zollner, Alois Jungbauer, Patricia Pereira Aguilar*

**Supplementary Material**


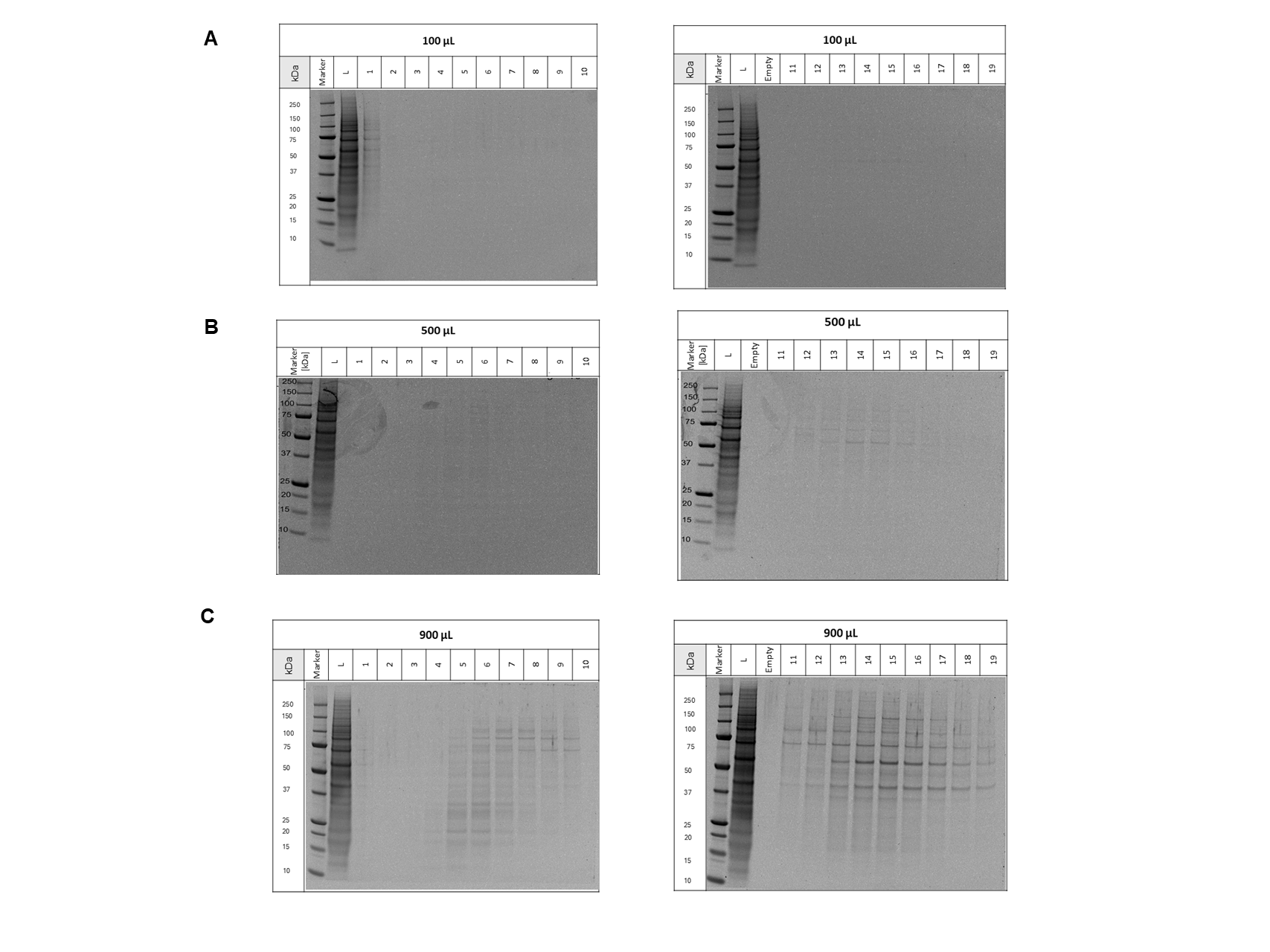


**Figure S1**: Flamingo stain analysis of AF4 collected fractions from injection volumes of 100 µL (A), 500 µL (B) and 900 µL (C) for detection of proteins with Flamingo Fluorescent Protein Gel Stain (Bio-Rad, Hercules, CA, USA). The SDS-PAGE gels were loaded with a size marker (Marker), the clarified cell culture supernatant containing HIV-1 gag VLPs (L) as well as the AF4 collected elution fractions (1-19).
